# Exploring adjunctive therapies for cerebral malaria

**DOI:** 10.3389/fcimb.2024.1347486

**Published:** 2024-02-12

**Authors:** Johanna Bensalel, Julio Gallego-Delgado

**Affiliations:** ^1^ Ph.D. Program in Biology, The Graduate Center, The City University of New York, New York, NY, United States; ^2^ Department of Biological Sciences, Lehman College, City University of New York, New York, NY, United States; ^3^ Ph.D. Program in Biochemistry, The Graduate Center, The City University of New York, New York, NY, United States

**Keywords:** cerebral malaria, severe malaria, adjunctive therapy, endothelium, child mortality

## Abstract

Cerebral malaria (CM) is one of the most severe complications of malaria infection characterized by coma and neurological effects. Despite standardized treatment of malaria infection with artemisinin-based combination therapies (ACT), the mortality rate is still high, and it primarily affects pediatric patients. ACT reduces parasitemia but fails to adequately target the pathogenic mechanisms underlying CM, including blood-brain-barrier (BBB) disruption, endothelial activation/dysfunction, and hyperinflammation. The need for adjunctive therapies to specifically treat this form of severe malaria is critical as hundreds of thousands of people continue to die each year from this disease. Here we present a summary of some potential promising therapeutic targets and treatments for CM, as well as some that have been tested and deemed ineffective or, in some cases, even deleterious. Further exploration into these therapeutic agents is warranted to assess the effectiveness of these potential treatments for CM patients.

## 1 Introduction

**Figure 1:**
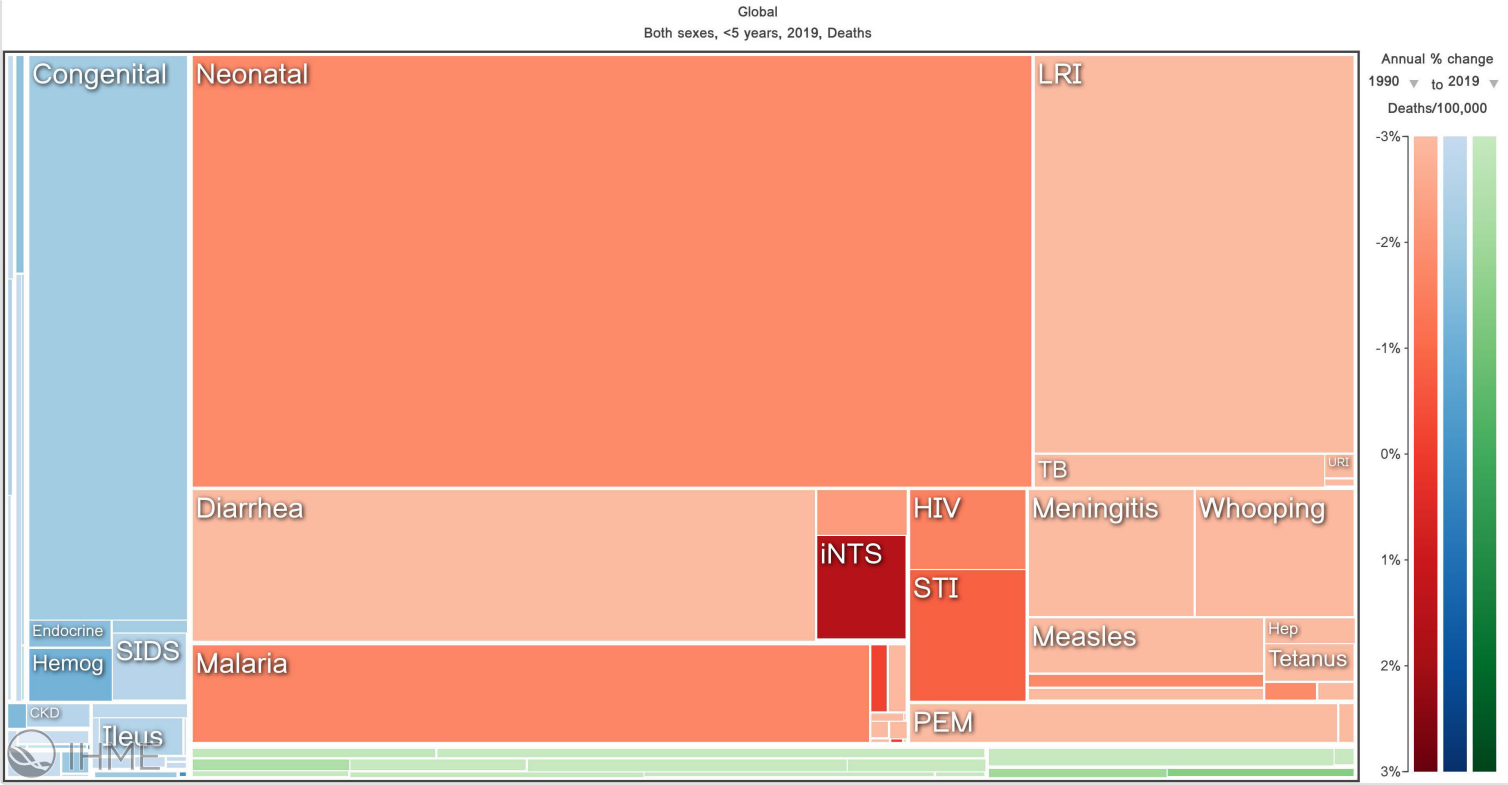
Visualization of major causes of child mortality across the globe. This figure illustrates that malaria is one of the top five causes of mortality in children under five years of age globally. The data shown is from 1990-2019. Obtained from Institute for Health Metrics and Evaluation (IHME). GBD Compare. Seattle, WA: IHME, University of Washington, 2015. Available from http://vizhub.healthdata.org/gbd-compare. (Accessed January 2, 2023).

It is estimated that in 2022 there were 249 million cases of malaria and 608,000 of those cases were fatal (World Health Organization, 2023). Most of these deaths occur in children under five years of age. In fact, malaria is one of the leading causes of child mortality (**Figure 1**), claiming the lives of approximately half a million children each year (Roser, 2022). The disease is caused by infection with a blood-borne pathogen of the *Plasmodium* species. There are five species that can infect humans; however, most cases of malaria are caused by infection with the species *Plasmodium falciparum*, which is prevalent in regions of sub-Saharan Africa (Sato, 2021). In many cases, infection will result in what is called uncomplicated malaria and can be treated relatively easily and effectively with a standard antimalarial regimen which consists of artemisinin-based combination therapy. In less than 2% of cases, however, patients develop what is called severe malaria in which the function of major organs or tissue systems is severely affected, and the risk of mortality greatly increases (Moxon et al., 2020).

Most severe malaria cases are caused by infection with the *P. falciparum* species and one of the most severe manifestations of this disease is cerebral malaria (CM), which is characterized primarily by impaired consciousness and/or coma (Schiess et al., 2020). Left untreated, this complication almost certainly results in death, often within 24 hours. Even with the aid of antimalarial drugs, the mortality rate is still as high as 20-30% (Song et al., 2022). Moreover, survivors of CM are prone to a host of often persistent neurological sequelae including seizures, language deficits, motor deficits, cognitive impairment, and other forms of neurological deficiency (Idro et al., 2006; Kariuki et al., 2014; Christensen and Eslick, 2015).

While the exact neuropathogenesis of CM remains unclear, what is clear is that CM patients suffer from a breakdown of the blood brain barrier (BBB) and neuronal and axonal injury (Dorovini-Zis et al., 2011). The BBB refers essentially to the vascular system that serves the central nervous system (CNS), comprised of brain microvascular endothelial cells (BMECs) attached to a basement membrane and supported by pericytes. BMECs interact with the surrounding neural and immune cells which are referred to as the neurovascular unit. The BBB isolates the CNS from the peripheral circulatory system to protect it from exposure to circulating pathogens or toxins, while simultaneously allowing for the bidirectional flow of necessary ions and compounds. BMECs are unique from peripheral endothelial cells in that they lack fenestrations and possess tight junctions, gap junctions, and adherens junctions to form a tightly bound monolayer with limited permeability and highly regulated transcellular transport (Profaci et al., 2020). Only molecules under 400 daltons can pass through this barrier (He et al., 2018). The tight junctions are closest to the apical surface and limit entry of solutes and ions from the bloodstream; these are made up of claudins, occludins, zona occludens protein-1 (ZO-1), and vinculin. Gap junctions form channels between BMECs to facilitate intercellular communication and maintain a tight connection between cells. Adherens junctions bind BMECs to the basement membrane and are made up of membrane-associated proteins including VE-cadherin, PECAM-1, and beta-catenin (Profaci et al., 2020).

In CM, expression of tight-junction-associated proteins including ZO-1, occludin, and vinculin is decreased. Expression of leukocyte adhesion molecules, such as ICAM-1, VCAM-1, and E-selectin, is also increased, when under normal conditions BMECs express these surface proteins at low levels. There is also an increase in BMEC secretion of pro-inflammatory and anti-inflammatory cytokines and chemokines (Dorovini-Zis et al., 2011). While it is still not completely understood, there are many factors in *falciparum* infection that potentially contribute to the disruption of the BBB. *Plasmodium falciparum*-infected red blood cells (*Pf*iRBCs) express a parasite-derived surface protein, PfEMP1, that confers the ability to adhere to endothelial cell surface receptors such as ICAM-1, EPCR, and CD36, to induce endothelial activation (Oquendo et al., 1989; Smith et al., 2000; Turner et al., 2013). Infected erythrocytes can also adhere to each other, as well as to non-infected erythrocytes, in a phenomenon known as rosetting. Rosetting and cytoadherence result in sequestration of *Pf*iRBCs in the vasculature system, obstructing blood flow. Additionally, rupture of *Pf*iRBCs, as part of the parasite’s natural blood stage cycle, results in the release of PAMPs and DAMPs that can bind both BMECs and other circulating immune cells. Parasite sequestration, release of *Pf*iRBC contents, and the ensuing inflammatory response synergistically induce endothelial activation and a loss of BMEC monolayer integrity (Moxon et al., 2020). The result is increased BBB permeability which can lead to brain swelling, intracranial hypertension, and hemorrhaging in CM (Idro et al., 2010).

It is important to note that the *Plasmodium* species used in rodent models of experimental CM (ECM), often *Plasmodium berghei* ANKA, does not induce cytoadherence of infected erythrocytes as in the case of *P. falciparum* infection. However, animals exhibit brain microvasculature obstruction, hyperinflammation, BBB disruption, and endothelial activation similarly to CM patients, making this a useful model for studying CM and exploring potential treatment options. The differences between the mechanisms of BBB disruption in CM and ECM have been reviewed elsewhere in greater detail (Albrecht-Schgoer et al., 2022).

### 1.1 Cerebral malaria: clinical features and management

Cerebral malaria is diagnosed when patients are in a comatose state that is not a cause of any other complications, such as coinfection with another pathogen, and asexual malaria parasites are detected in peripheral blood, which is detected by preparing a peripheral blood smear. Convulsions and retinal changes are also common in CM patients. Aside from the timely intravenous administration of antimalarials, there are no specific treatments for the underlying causes of CM. Fever management is suggested and patients in coma must be monitored and provided supportive care to manage symptoms. Convulsions can be treated with slow intravenous administration of benzodiazepine, and in more severe cases, phenytoin or, as a last resort, phenobarbitone (World Health Organization, 2012; Taylor and Molyneux, 2015). Mechanical ventilatory support are critical but they are not readily available in many areas where malaria is endemic (Albrecht-Schgoer et al., 2022). In approximately 20-30% of cases, the current management techniques are insufficient and result in death (Song et al., 2022). It is clear that there is an urgent need for specific adjunctive therapies to treat the underlying causes of CM and prevent fatalities.

### 1.2 Adjunctive treatments demonstrated to be ineffective in CM

Phenobarbital is a drug normally used to treat seizures. In one clinical trial, administration of intramuscular phenobarbital to CM patients significantly reduced incidence of seizure. However, in a later study with pediatric CM patients, phenobarbital effectively prevented seizures but increased the mortality rate as well as the frequency of respiratory arrest (Crawley et al., 2000). For this reason, phenobarbital is not recommended to treat CM patients suffering from seizures unless first line treatments of benzodiazepine or phenytoin are not effective, and even then, patients’ respiratory activity must be monitored closely (World Health Organization, 2012).

Mannitol therapy is often used to reduce intracranial pressure from brain swelling in other disease contexts. In trials with pediatric CM patients, however, mannitol did not significantly impact clinical outcomes and in a randomized trial with adult CM patients, mannitol proved ineffective in reducing swelling and resulted in increased mortality rates and longer coma durations (Namutangula et al., 2007; Mohanty et al., 2011).

Platelet accumulation in the brain microvasculature has been observed in post-mortem tissue of CM patients that did not survive (Grau et al., 2003). Heparin and acetylsalicylic acid (ASA; commonly known as aspirin), which are normally used to treat blood clots and fever, were used to treat severe falciparum malaria patients. However, neither heparin nor ASA improved clinical outcomes in patients assessed by parasite clearance rate, fever alleviation, or length of hospitalization (Hemmer et al., 1991).

Dexamethasone is a corticosteroid that is often used to relieve inflammation. In several clinical trials, dexamethasone was used to treat CM patients but was not effective in reducing neurological deficits or improving survival when compared to the control groups, and in one trial, it even conferred deleterious effects (Warrell et al., 1982 **;** Hoffman et al., 1988 **;** Prasad and Garner, 2000).

TNF-alpha is upregulated in CM, however, modulating levels of TNF-alpha through anti-TNF monoclonal antibody treatment did not increase survival in a trial with pediatric CM patients and was correlated with increased neurological sequelae (van Hensbroek et al., 1996). Pentoxifylline is a phosphodiesterase inhibitor that reduces TNF levels and proved promising in two clinical trials with adult and pediatric patients of CM based on increased survival and shorter coma duration (Di Perri et al., 1995; Das et al., 2003). However, in another trial conducted with falciparum malaria patients, treatment with pentoxifylline did not have any effect on clinical outcome and patients reported mild adverse side effects, while in one trial with a small sample size of ten pediatric CM patients, the mortality rate was unusually high in the pentoxifylline-treated group compared to the control group (Hemmer et al., 1997; Lell et al., 2010). Intravenous administration of immunoglobulin was also shown not to be superior to placebo in a trial with pediatric CM patients and may have been associated with deleterious effects (Taylor et al., 1992).

### 1.3 Potential adjunctive therapies for CM

Immunomodulation has become a popular method of disease intervention in recent years. In CM, the inflammatory response, initiated by release of DAMPs and PAMPs upon PfiRBC rupture and then bolstered by endothelial activation, can create a hyperinflammatory environment that can have deleterious effects. Inhibiting T-cell metabolism and proliferation via administration of Gln analog 6-diazo-5-oxo-l-norleucine (DON) improved survival rates in mice with ECM when administered after presentation of neurological signs of CM. Moreover, DON treatment improved neurological outcomes, reduced brain swelling, and helped recover the BBB integrity in these mice (Gordon et al., 2015). The efficacy of DON as an adjunctive treatment for CM is currently being determined in clinical trials (**Table 1**).

**Table 1:** List of potential adjunctive treatments for CM.

Treatment	Class of drug/mode of action	ECM study reference	Clinical trial status
Ang1 protein	Restores homeostatic levels of angiopoietin 1 and 2	Higgins et al., 2016	
Artemisone	Antimalarial and anti-inflammatory	Golenser et al., 2020	
AT1 blockers Irbesartan, losartan	Angiotensin II receptor modulator	Gallego-Delgado et al., 2016 Mota et al., 2022	
AT2 agonists Compound 2, CGP-42112A	Angiotensin II receptor modulator	Gallego-Delgado et al., 2016	
Atorvastatin	Statin; regulates angiopoietin 1 and 2	Mota et al., 2022; Wilson et al., 2013	
Curcumin	Anti-inflammatory; reduced endothelial cell apoptosis and restored endothelial barrier integrity	Waknine-Grinberg et al., 2010	
Erythropoietin	Immunomodulation; inhibits function of splenic dendritic cells and promotes increased circulation of regulatory T cells	Wei et al., 2014	Status uknown (NCT00697164)
Fasudil	Rho kinase inhibitor; reduces endothelial cell apoptosis and restores endothelial barrier integrity	Waknine-Grinberg et al., 2010	
Gln analog 6-diazo-5-oxo-l-norleucine (DON)	Immunomodulation; inhibits T cell expansion/metabolism	Gordon et al., 2015	Phase I/II (NCT05478720)
Intravenous hypertonic saline	Reduces brain swelling	Gad et al., 2018	Phase III (NCT03300648)
Lovastatin	Statin; regulates angiopoietin 1 and 2	Reis et al., 2012	
Neuregulin-1	Protects cells from apoptosis and reduces disruption of BBB	Liu et al., 2018	
Oral activated charcoal (OAC)	Immunomodulation; reduces proinflammatory cytokines	de Souza et al., 2010	
Rosigliatazone	Peroxisome proliferator-activated receptor-γ (PPARγ) agonist; neuroprotective and anti-inflammatory	Serghides et al., 2014	
Sphingosine-1 phosphate (S1P)	Sphingolipid; G-protein coupled receptor ligand that regulates brain endothelial cell homeostasis	Finney et al., 2011	
Vitamin D	Steroid hormone; protects BBB integrity, reduced endothelial activation markers and inflammation markers	Dwivedi et al., 2016	

There are other promising agents such as oral activated charcoal which was shown to reduce pro-inflammatory cytokine levels and improve survival in a murine model of ECM. When administered to healthy humans in a phase 1 clinical trial, it did not interfere with the pharmacokinetics of artesunate (de Souza et al., 2010). While potentially an effective treatment, oral activated charcoal however, must be administered to infants and young children in a comatose state through a nasogastric tube which may not always be available in resource-limited areas where malaria is endemic. In a murine model of CM, administration of recombinant human erythropoietin successfully attenuated inflammation by inhibiting the function of splenic dendritic cells and promoting increased circulation of regulatory T cells (Wei et al., 2014). In another study, artemisone, a relatively new artemisinin derivative, was administered to mice with ECM via subcutaneous injection of a pasty polymer form of the drug. This method of administration ensured a slow release of the drug and was given prophylactically. The result was reduced parasitemias and improved survival of mice that were treated versus those that were not. TNF levels were also reduced while anti-inflammatory cytokines IL-4 and IL-10 were increased in artemisone-treated mice. The treatment also counteracted BBB disruption, making it a potential candidate for clinical trials (Golenser et al., 2020).

Endothelial activation/dysfunction plays a major role in CM pathogenesis and is a therapeutic target worth consideration (Kim et al., 2011). Beta-catenin, one of the proteins that form the adherens junctions in BMECs, is activated in response to *Pf*iRBC rupture *in vitro*. Beta-catenin is regulated by the Angiotensin II receptors type 1 and 2 (AT1 and AT2). In a murine model of ECM, administration of irbesartan, an AT1 inhibitor, or Compound 21, an AT2 agonist, in combination with the antimalarial drug chloroquine, rescued around 60% of the mice with critical signs of CM compared to 14% of mice treated with chloroquine alone. These, and other modulators of the AT1/2 receptors such as losartan or CGP-42112A, may be promising adjunctive therapies that have yet to be tested in clinical trials (Gallego-Delgado et al., 2016).

The angiopoietin (Ang)-Tie2 axis is another important regulator of endothelial activation and potential therapeutic target. Ang1 and Ang2 are competitive ligands of the tyrosine kinase receptor Tie2, which is expressed on endothelial cells. Under normal conditions, Ang1 concentrations greatly surpass those of Ang 2 so that Ang1 binds Tie2, which phosphorylates AKT to prevent BMEC apoptosis and maintain homeostatic activity. In CM, Ang2 concentrations increase and prevent Ang1 from binding Tie2, ultimately resulting in weakening of intercellular junctions and expression of leukocyte adhesion molecules ICAM1 and VCAM1(Sack et al., 2020). Administration of Ang1 protein in combination with artesunate increased survival in mice with ECM, compared to mice treated with artesunate alone (Higgins et al., 2016). Additionally, inhibition of Ang2 binding using an Ang2 antibody treatment significantly reduced mortality in a murine model of sepsis in which endothelial activation also contributes to pathogenesis (Ziegler et al., 2013). In another sepsis murine model, an Ang2 antibody that could both neutralize Ang2 and activate Tie2 greatly ameliorated disease progression (Han et al., 2016). Restoring the ratio of Ang1:Ang2 activity to improve disease outcome has been successfully demonstrated in the context of other infections in which endothelial activation and BBB disruption contributes to the pathogenesis, such as anthrax, Ebola hemorrhagic fever, and dengue fever (Ghosh et al., 2012; Rasmussen et al., 2014; Phanthanawiboon et al., 2016).

Rosigliatazone is a peroxisome proliferator-activated receptor-γ (PPARγ) agonist used to treat type 2 diabetes mellitus. Treatment with rosigliatazone and artesunate together resulted in increased levels of circulating Ang1 and decreased Ang2 transcription in the brains of mice with ECM, as compared to control mice treated with only artesunate. Similarly, human patients with non-severe falciparum malaria that were treated with rosigliatazone and artesunate combination therapy were found to have a lower plasma Ang2:Ang1 ratio in (Serghides et al., 2014). This drug has also been demonstrated to induce secretion of neuroprotective brain-derived neurotrophic factor (BDNF), improve neurocognitive outcomes, and reduce biomarkers of inflammation in non-severe malaria (Boggild et al., 2009; Serghides et al., 2014). Rosigliatazone may be a promising candidate for adjuvant treatment of CM.

Statins are drugs currently approved for use to treat high cholesterol and in a few studies have been shown to improve outcomes from ECM, though the exact mechanism of action is not completely understood. Treatment with a combination of atorvastatin and irbesartan, an angiotensin II receptor inhibitor clinically used to treat hypertension, in combination with antimalarial drugs significantly increased survival rates of mice with ECM when compared to mice treated with antimalarial drugs alone. Moreover, this combination treatment reduced the quantity and size of brain hemorrhages, as well as, levels of plasma biomarkers of endothelial activation, including soluble Ang1 (Mota et al., 2022). Atorvastatin treatment has been demonstrated to increase Ang1 transcription and decrease Ang2 expression in the brain tissue of mice with ECM (Wilson et al., 2013). Another statin that has proven effective in reducing mortality and improving neurocognitive outcomes in a murine model of ECM is lovastatin. Edema, ICAM-1, and CD11b mRNA levels were reduced in brain tissue of mice treated with lovastatin in combination with antimalarial drugs (Reis et al., 2012).

Sphingosine-1 phosphate (S1P) is a sphingolipid that acts as a signaling molecule via G-protein coupled receptors S1P1-5 to regulate BMEC homeostasis, and it has been implicated in CM. Fingolimod is an S1P receptor modulator that regulates BMEC intercellular adhesion by stabilizing VE-cadherin. In a murine model of ECM, fingolimod, in combination with artesunate, significantly improved survival rates, reduced levels of soluble ICAM, increased Ang1 levels, and decreased BBB permeability when compared to mice treated with artesunate alone (Finney et al., 2011). Fingolimod is already approved to treat multiple sclerosis and may be a promising candidate for trials with CM patients.

Rho kinase activation is another mechanism that has been implicated in BBB disruption and brain edema; furthermore, it has been demonstrated that *Pf*iRBC adhesion to endothelial cells stimulates Rho signaling *in vitro*. Rho kinase inhibitor, Fasudil, reduced endothelial cell apoptosis and restored endothelial barrier integrity after exposure to *Pf*iRBCs *in vitro* (Taoufiq et al., 2008). In another study, Fasudil either completely prevented or delayed the onset of ECM in mice injected with Plasmodium berghei ANKA, and those mice treated with Fasudil exhibited significantly higher survival rates compared to the untreated controls. Interestingly, in the same study, curcumin treatment had a similar effect (Waknine-Grinberg et al., 2010).Fasudil is a drug that is already in clinical use that is used to treat brain ischemia and cerebral vasospasm.

In a similar fashion, neuregulin-1 protects BMECs from apoptosis and reduces disruption of BBB integrity in a model of heme-induced BBB disruption *in vitro*. Moreover, in a murine model of ECM, neuregulin-1 treatment reduced mortality by stimulating ErbB4 phosphorylation which ultimately leads to AKT activation (Liu et al., 2018). In another study of ECM, the use of vitamin D co-administered with intramuscular arteether (an ethyl ether derivative of artemisinin) significantly improved survival rates when compared to untreated mice or mice that were treated with either vitamin D or arteether alone. BBB integrity was also protected and levels of endothelial activation markers ICAM-1 and VCAM-1, as well as inflammation markers, were greatly reduced (Dwivedi et al., 2016).

## 3 Discussion

The toll of CM is immense in countries where *P. falciparum* infection is prevalent and/or endemic. The effects are not only evident in the drastic child mortality rate but also in the potential long-term effects seen thereafter, including neurological disorders and impairments, and often persistent endothelial activation (Idro et al., 2006 **;** Kariuki et al., 2014 **;** Moxon et al., 2014 **;** Christensen and Eslick, 2015). Additionally, CM often increases a pediatric patient’s risk for comorbidities such as respiratory distress and acute kidney injury, which have their own sets of challenges and sequelae (Burton, 2017 **;** Conroy et al., 2023). As of now, the only approved treatment for CM is the use of antimalarial drugs to reduce and eliminate parasitemia, but in up to 20-30% of the cases, this is not enough. Here we have presented a summary of some of the treatments that seem most promising with a focus on those that target endothelial dysfunction and BBB disruption, which are primary contributors to CM pathogenesis. Some of these treatments are already approved for clinical use in other disease contexts or are currently in clinical trials for the treatment of CM. Many have not progressed to clinical trials but have been tested in ECM models. As noted earlier, in ECM, infected erythrocytes are not cytoadherent as in *P. falciparum* infection. However, although ECM presents significant limitations in terms of precise replication of CM in humans, the model still provides valuable insight into the molecular pathogenesis of the disease and is an important tool in identifying potential therapeutic targets that should be considered for clinical testing (Ghazanfari et al., 2018). Nonetheless, not all treatments that have proven successful in animal models of ECM have demonstrated efficacy in clinical trials. To address this, we need to develop more physiological models of CM, either with better animal models that reproduce more precisely the pathogenic mechanisms in humans or using *in vitro* models that accurately reproduce the BBB microenvironment, such as 3D microchips fabricated to facilitate coculture of brain endothelial cells and astrocytes or pericytes or the use of organoid arrays (Herland et al., 2016; Simonneau et al., 2021).The potential therapies presented here may prove effective and safe, yet additional research is required to determine the most suitable candidates for adjunctive treatment in both pediatric and adult patients of CM. An effective adjunctive treatment for CM could save hundreds of thousands of lives each year, prevent long-term neurological sequelae in CM patients, and reduce the socio-economic burden of this disease in countries where malaria is endemic.

## Author contributions

JB: Writing – original draft, Writing – review & editing. JG-D: Writing – original draft, Writing – review & editing.
